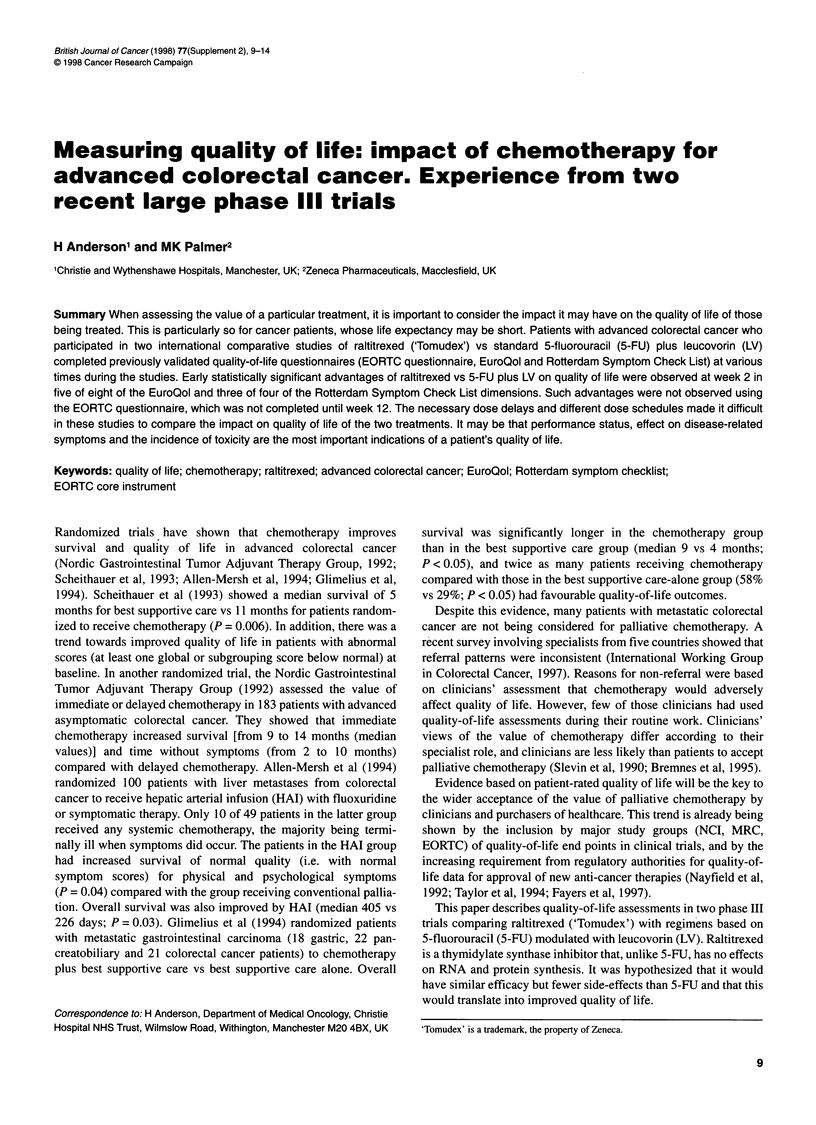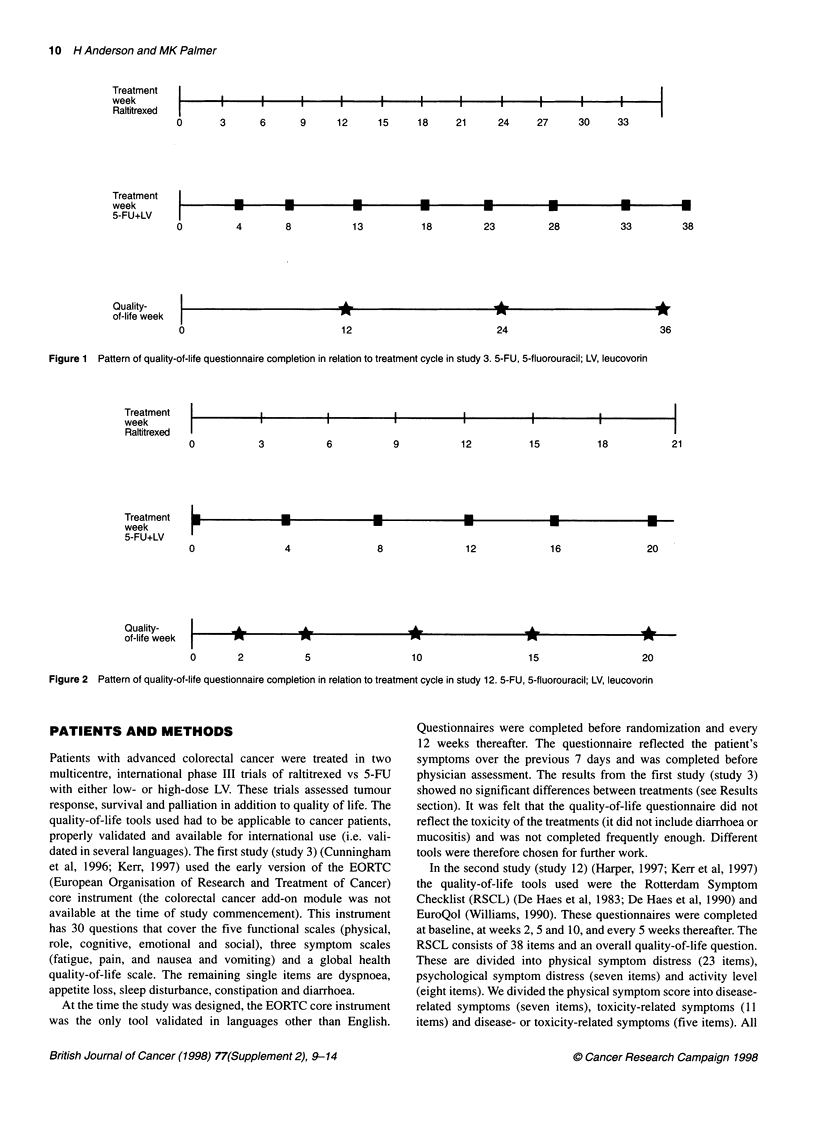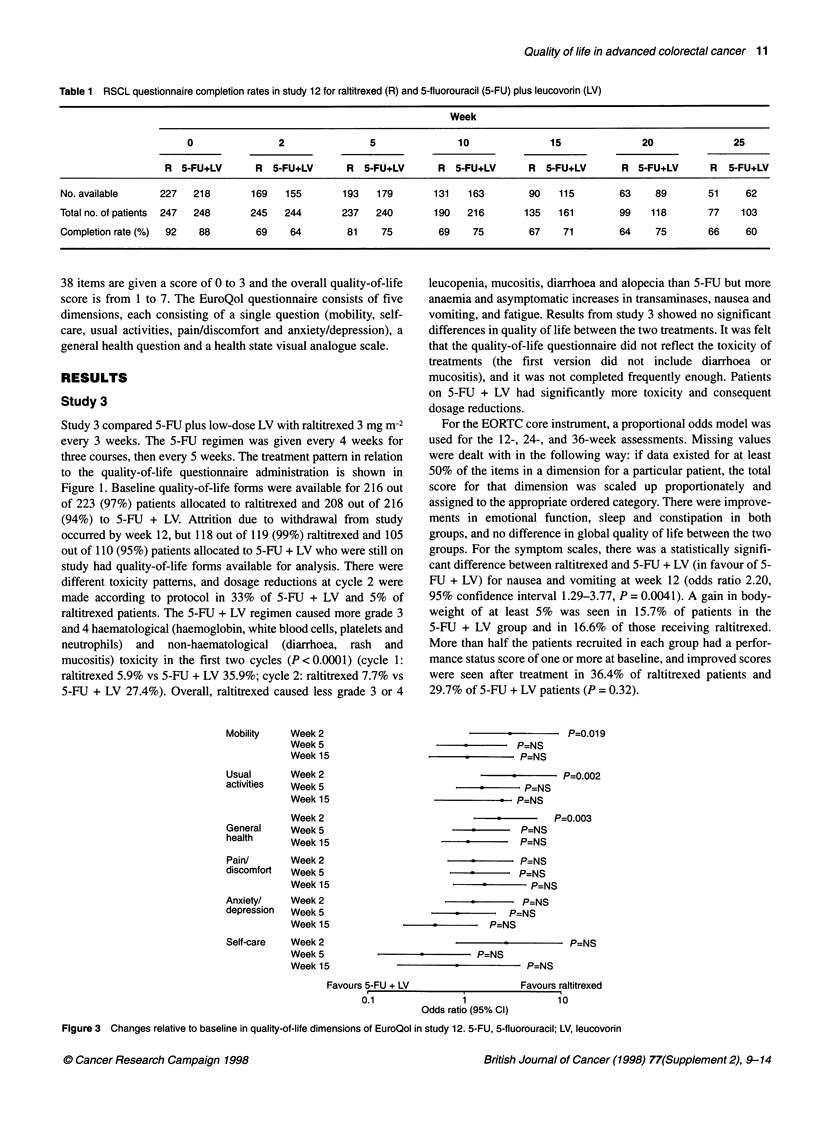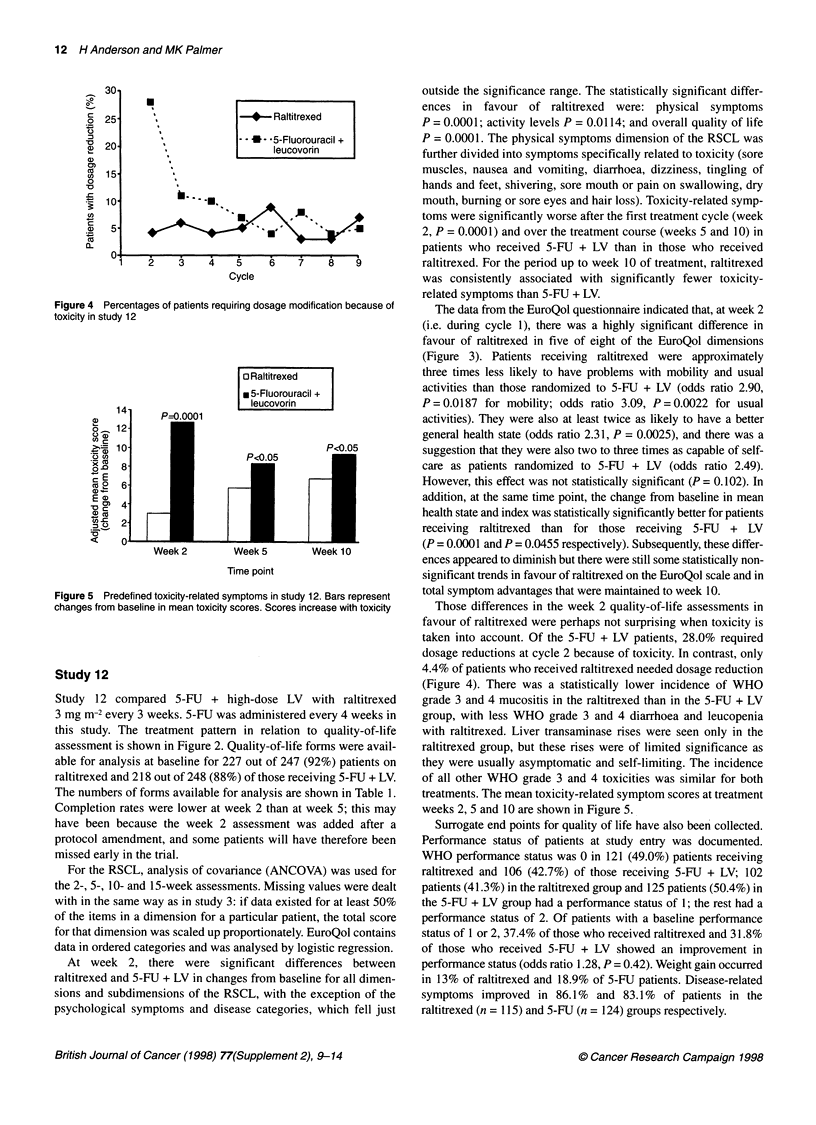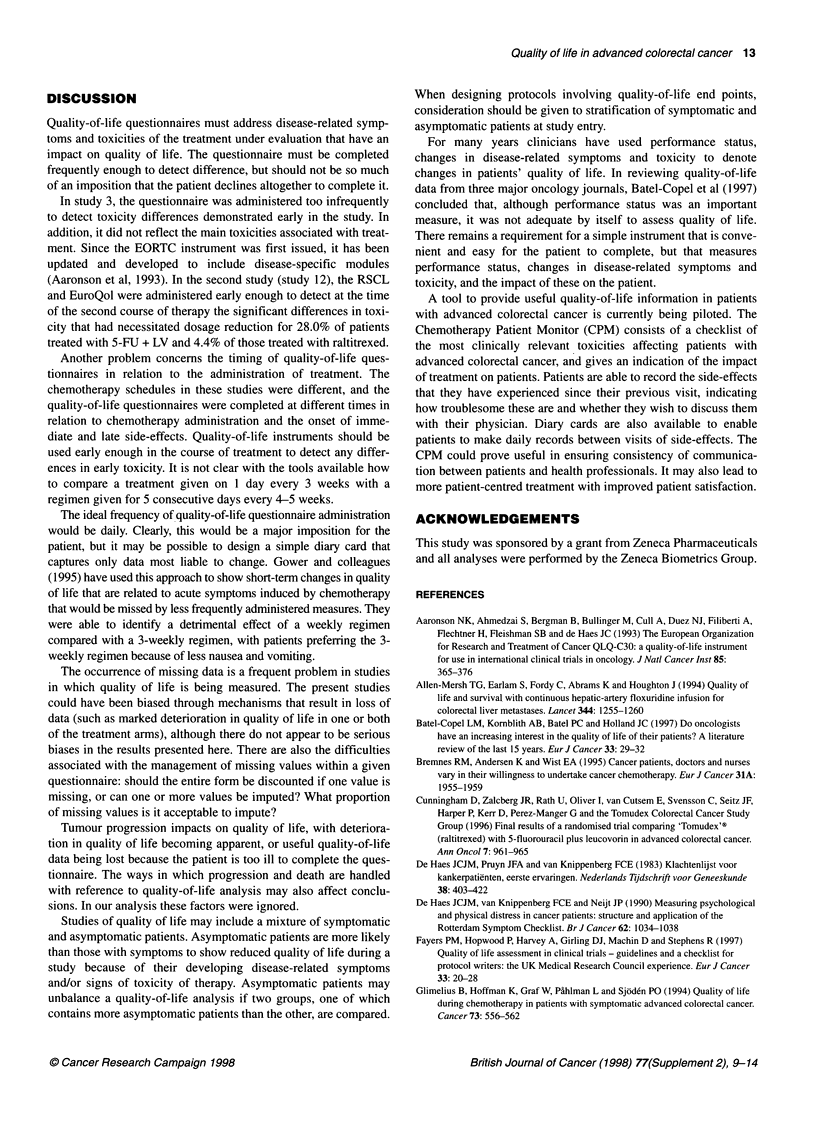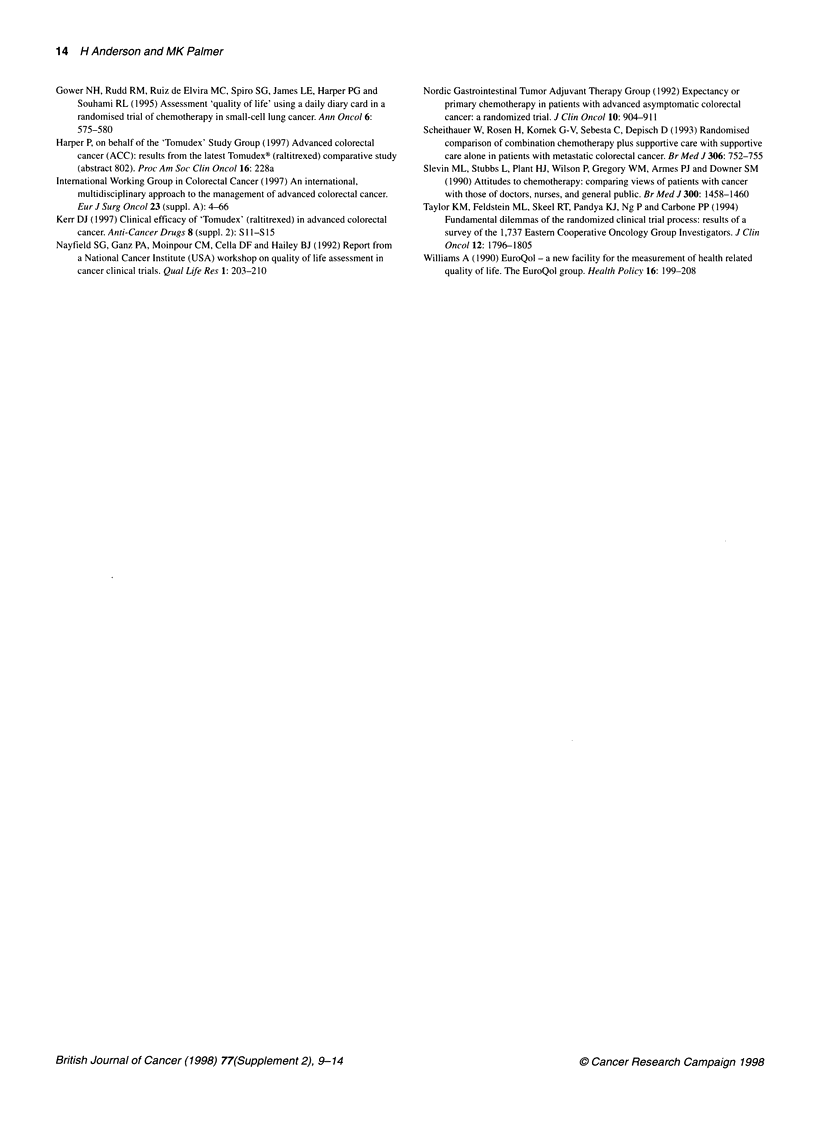# Measuring quality of life: impact of chemotherapy for advanced colorectal cancer. Experience from two recent large phase III trials.

**DOI:** 10.1038/bjc.1998.420

**Published:** 1998

**Authors:** H. Anderson, M. K. Palmer

**Affiliations:** Christie Hospital, Withington, Manchester, UK.

## Abstract

When assessing the value of a particular treatment, it is important to consider the impact it may have on the quality of life of those being treated. This is particularly so for cancer patients, whose life expectancy may be short. Patients with advanced colorectal cancer who participated in two international comparative studies of raltitrexed ('Tomudex') vs standard 5-fluorouracil (5-FU) plus leucovorin (LV) completed previously validated quality-of-life questionnaires (EORTC questionnaire, EuroQol and Rotterdam Symptom Check List) at various times during the studies. Early statistically significant advantages of raltitrexed vs 5-FU plus LV on quality of life were observed at week 2 in five of eight of the EuroQol and three of four of the Rotterdam Symptom Check List dimensions. Such advantages were not observed using the EORTC questionnaire, which was not completed until week 12. The necessary dose delays and different dose schedules made it difficult in these studies to compare the impact on quality of life of the two treatments. It may be that performance status, effect on disease-related symptoms and the incidence of toxicity are the most important indications of a patient's quality of life.


British Journal of Cancer (1998) 77(Supplement 2), 9–14
© 1998 Cancer Research Campaign

### Measuring quality of life: impact of chemotherapy for advanced colorectal cancer. Experience from two recent large phase III trials

**H Anderson[1] and MK Palmer[2]**

[1]Christie and Wythenshawe Hospitals, Manchester, UK; [2]Zeneca Pharmaceuticals, Macclesfield, UK

**Summary** When assessing the value of a particular treatment, it is important to consider the impact it may have on the quality of life of those being treated. This is particularly so for cancer patients, whose life expectancy may be short. Patients with advanced colorectal cancer who participated in two international comparative studies of raltitrexed ('Tomudex') vs standard 5-fluorouracil (5-FU) plus leucovorin (LV) completed previously validated quality-of-life questionnaires (EORTC questionnaire, EuroQol and Rotterdam Symptom Check List) at various times during the studies. Early statistically significant advantages of raltitrexed vs 5-FU plus LV on quality of life were observed at week 2 in five of eight of the EuroQol and three of four of the Rotterdam Symptom Check List dimensions. Such advantages were not observed using the EORTC questionnaire, which was not completed until week 12. The necessary dose delays and different dose schedules made it difficult in these studies to compare the impact on quality of life of the two treatments. It may be that performance status, effect on disease-related symptoms and the incidence of toxicity are the most important indications of a patient's quality of life.

**Keywords:** quality of life; chemotherapy; raltitrexed; advanced colorectal cancer; EuroQol; Rotterdam symptom checklist; EORTC core instrument

Randomized trials have shown that chemotherapy improves survival and quality of life in advanced colorectal cancer (Nordic Gastrointestinal Tumor Adjuvant Therapy Group, 1992; Scheithauer et al, 1993; Allen-Mersh et al, 1994; Glimelius et al, 1994). Scheithauer et al (1993) showed a median survival of 5 months for best supportive care vs 11 months for patients randomized to receive chemotherapy ($P = 0.006$). In addition, there was a trend towards improved quality of life in patients with abnormal scores (at least one global or subgrouping score below normal) at baseline. In another randomized trial, the Nordic Gastrointestinal Tumor Adjuvant Therapy Group (1992) assessed the value of immediate or delayed chemotherapy in 183 patients with advanced asymptomatic colorectal cancer. They showed that immediate chemotherapy increased survival [from 9 to 14 months (median values)] and time without symptoms (from 2 to 10 months) compared with delayed chemotherapy. Allen-Mersh et al (1994) randomized 100 patients with liver metastases from colorectal cancer to receive hepatic arterial infusion (HAI) with fluoxuridine or symptomatic therapy. Only 10 of 49 patients in the latter group received any systemic chemotherapy, the majority being terminally ill when symptoms did occur. The patients in the HAI group had increased survival of normal quality (i.e. with normal symptom scores) for physical and psychological symptoms ($P = 0.04$) compared with the group receiving conventional palliation. Overall survival was also improved by HAI (median 405 vs 226 days; $P = 0.03$). Glimelius et al (1994) randomized patients with metastatic gastrointestinal carcinoma (18 gastric, 22 pancreatobiliary and 21 colorectal cancer patients) to chemotherapy plus best supportive care vs best supportive care alone. Overall survival was significantly longer in the chemotherapy group than in the best supportive care group (median 9 vs 4 months; $P < 0.05$), and twice as many patients receiving chemotherapy compared with those in the best supportive care-alone group (58% vs 29%; $P < 0.05$) had favourable quality-of-life outcomes.

Despite this evidence, many patients with metastatic colorectal cancer are not being considered for palliative chemotherapy. A recent survey involving specialists from five countries showed that referral patterns were inconsistent (International Working Group in Colorectal Cancer, 1997). Reasons for non-referral were based on clinicians' assessment that chemotherapy would adversely affect quality of life. However, few of those clinicians had used quality-of-life assessments during their routine work. Clinicians' views of the value of chemotherapy differ according to their specialist role, and clinicians are less likely than patients to accept palliative chemotherapy (Slevin et al, 1990; Bremnes et al, 1995).

Evidence based on patient-rated quality of life will be the key to the wider acceptance of the value of palliative chemotherapy by clinicians and purchasers of healthcare. This trend is already being shown by the inclusion by major study groups (NCI, MRC, EORTC) of quality-of-life end points in clinical trials, and by the increasing requirement from regulatory authorities for quality-of-life data for approval of new anti-cancer therapies (Nayfield et al, 1992; Taylor et al, 1994; Fayers et al, 1997).

This paper describes quality-of-life assessments in two phase III trials comparing raltitrexed ('Tomudex') with regimens based on 5-fluorouracil (5-FU) modulated with leucovorin (LV). Raltitrexed is a thymidylate synthase inhibitor that, unlike 5-FU, has no effects on RNA and protein synthesis. It was hypothesized that it would have similar efficacy but fewer side-effects than 5-FU and that this would translate into improved quality of life.

*Correspondence to:* H Anderson, Department of Medical Oncology, Christie Hospital NHS Trust, Wilmslow Road, Withington, Manchester M20 4BX, UK

'Tomudex' is a trademark, the property of Zeneca.

**Figure 1** Pattern of quality-of-life questionnaire completion in relation to treatment cycle in study 3. 5-FU, 5-fluorouracil; LV, leucovorin

**Figure 2** Pattern of quality-of-life questionnaire completion in relation to treatment cycle in study 12. 5-FU, 5-fluorouracil; LV, leucovorin

### PATIENTS AND METHODS

Patients with advanced colorectal cancer were treated in two multicentre, international phase III trials of raltitrexed vs 5-FU with either low- or high-dose LV. These trials assessed tumour response, survival and palliation in addition to quality of life. The quality-of-life tools used had to be applicable to cancer patients, properly validated and available for international use (i.e. validated in several languages). The first study (study 3) (Cunningham et al, 1996; Kerr, 1997) used the early version of the EORTC (European Organisation of Research and Treatment of Cancer) core instrument (the colorectal cancer add-on module was not available at the time of study commencement). This instrument has 30 questions that cover the five functional scales (physical, role, cognitive, emotional and social), three symptom scales (fatigue, pain, and nausea and vomiting) and a global health quality-of-life scale. The remaining single items are dyspnoea, appetite loss, sleep disturbance, constipation and diarrhoea.

At the time the study was designed, the EORTC core instrument was the only tool validated in languages other than English. Questionnaires were completed before randomization and every 12 weeks thereafter. The questionnaire reflected the patient's symptoms over the previous 7 days and was completed before physician assessment. The results from the first study (study 3) showed no significant differences between treatments (see Results section). It was felt that the quality-of-life questionnaire did not reflect the toxicity of the treatments (it did not include diarrhoea or mucositis) and was not completed frequently enough. Different tools were therefore chosen for further work.

In the second study (study 12) (Harper, 1997; Kerr et al, 1997) the quality-of-life tools used were the Rotterdam Symptom Checklist (RSCL) (De Haes et al, 1983; De Haes et al, 1990) and EuroQol (Williams, 1990). These questionnaires were completed at baseline, at weeks 2, 5 and 10, and every 5 weeks thereafter. The RSCL consists of 38 items and an overall quality-of-life question. These are divided into physical symptom distress (23 items), psychological symptom distress (seven items) and activity level (eight items). We divided the physical symptom score into disease-related symptoms (seven items), toxicity-related symptoms (11 items) and disease- or toxicity-related symptoms (five items). All

© Cancer Research Campaign 1998

**Table 1** RSCL questionnaire completion rates in study 12 for raltitrexed (R) and 5-fluorouracil (5-FU) plus leucovorin (LV)

| | Week | | | | | | | | | | | | | |
|---|---|---|---|---|---|---|---|---|---|---|---|---|---|---|
| | 0 | | 2 | | 5 | | 10 | | 15 | | 20 | | 25 | |
| | R | 5-FU+LV | R | 5-FU+LV | R | 5-FU+LV | R | 5-FU+LV | R | 5-FU+LV | R | 5-FU+LV | R | 5-FU+LV |
| No. available | 227 | 218 | 169 | 155 | 193 | 179 | 131 | 163 | 90 | 115 | 63 | 89 | 51 | 62 |
| Total no. of patients | 247 | 248 | 245 | 244 | 237 | 240 | 190 | 216 | 135 | 161 | 99 | 118 | 77 | 103 |
| Completion rate (%) | 92 | 88 | 69 | 64 | 81 | 75 | 69 | 75 | 67 | 71 | 64 | 75 | 66 | 60 |

38 items are given a score of 0 to 3 and the overall quality-of-life score is from 1 to 7. The EuroQol questionnaire consists of five dimensions, each consisting of a single question (mobility, self-care, usual activities, pain/discomfort and anxiety/depression), a general health question and a health state visual analogue scale.

### RESULTS

### Study 3

Study 3 compared 5-FU plus low-dose LV with raltitrexed 3 mg $m^{-2}$ every 3 weeks. The 5-FU regimen was given every 4 weeks for three courses, then every 5 weeks. The treatment pattern in relation to the quality-of-life questionnaire administration is shown in Figure 1. Baseline quality-of-life forms were available for 216 out of 223 (97%) patients allocated to raltitrexed and 208 out of 216 (94%) to 5-FU + LV. Attrition due to withdrawal from study occurred by week 12, but 118 out of 119 (99%) raltitrexed and 105 out of 110 (95%) patients allocated to 5-FU + LV who were still on study had quality-of-life forms available for analysis. There were different toxicity patterns, and dosage reductions at cycle 2 were made according to protocol in 33% of 5-FU + LV and 5% of raltitrexed patients. The 5-FU + LV regimen caused more grade 3 and 4 haematological (haemoglobin, white blood cells, platelets and neutrophils) and non-haematological (diarrhoea, rash and mucositis) toxicity in the first two cycles ($P < 0.0001$) (cycle 1: raltitrexed 5.9% vs 5-FU + LV 35.9%; cycle 2: raltitrexed 7.7% vs 5-FU + LV 27.4%). Overall, raltitrexed caused less grade 3 or 4 leucopenia, mucositis, diarrhoea and alopecia than 5-FU but more anaemia and asymptomatic increases in transaminases, nausea and vomiting, and fatigue. Results from study 3 showed no significant differences in quality of life between the two treatments. It was felt that the quality-of-life questionnaire did not reflect the toxicity of treatments (the first version did not include diarrhoea or mucositis), and it was not completed frequently enough. Patients on 5-FU + LV had significantly more toxicity and consequent dosage reductions.

For the EORTC core instrument, a proportional odds model was used for the 12-, 24-, and 36-week assessments. Missing values were dealt with in the following way: if data existed for at least 50% of the items in a dimension for a particular patient, the total score for that dimension was scaled up proportionately and assigned to the appropriate ordered category. There were improvements in emotional function, sleep and constipation in both groups, and no difference in global quality of life between the two groups. For the symptom scales, there was a statistically significant difference between raltitrexed and 5-FU + LV (in favour of 5-FU + LV) for nausea and vomiting at week 12 (odds ratio 2.20, 95% confidence interval 1.29–3.77, $P = 0.0041$). A gain in body-weight of at least 5% was seen in 15.7% of patients in the 5-FU + LV group and in 16.6% of those receiving raltitrexed. More than half the patients recruited in each group had a performance status score of one or more at baseline, and improved scores were seen after treatment in 36.4% of raltitrexed patients and 29.7% of 5-FU + LV patients ($P = 0.32$).

**Figure 3** Changes relative to baseline in quality-of-life dimensions of EuroQol in study 12. 5-FU, 5-fluorouracil; LV, leucovorin

**Figure 4** Percentages of patients requiring dosage modification because of toxicity in study 12

**Figure 5** Predefined toxicity-related symptoms in study 12. Bars represent changes from baseline in mean toxicity scores. Scores increase with toxicity

### Study 12

Study 12 compared 5-FU + high-dose LV with raltitrexed 3 mg $m^{-2}$ every 3 weeks. 5-FU was administered every 4 weeks in this study. The treatment pattern in relation to quality-of-life assessment is shown in Figure 2. Quality-of-life forms were available for analysis at baseline for 227 out of 247 (92%) patients on raltitrexed and 218 out of 248 (88%) of those receiving 5-FU + LV. The numbers of forms available for analysis are shown in Table 1. Completion rates were lower at week 2 than at week 5; this may have been because the week 2 assessment was added after a protocol amendment, and some patients will have therefore been missed early in the trial.

For the RSCL, analysis of covariance (ANCOVA) was used for the 2-, 5-, 10- and 15-week assessments. Missing values were dealt with in the same way as in study 3: if data existed for at least 50% of the items in a dimension for a particular patient, the total score for that dimension was scaled up proportionately. EuroQol contains data in ordered categories and was analysed by logistic regression.

At week 2, there were significant differences between raltitrexed and 5-FU + LV in changes from baseline for all dimensions and subdimensions of the RSCL, with the exception of the psychological symptoms and disease categories, which fell just outside the significance range. The statistically significant differences in favour of raltitrexed were: physical symptoms $P = 0.0001$; activity levels $P = 0.0114$; and overall quality of life $P = 0.0001$. The physical symptoms dimension of the RSCL was further divided into symptoms specifically related to toxicity (sore muscles, nausea and vomiting, diarrhoea, dizziness, tingling of hands and feet, shivering, sore mouth or pain on swallowing, dry mouth, burning or sore eyes and hair loss). Toxicity-related symptoms were significantly worse after the first treatment cycle (week 2, $P = 0.0001$) and over the treatment course (weeks 5 and 10) in patients who received 5-FU + LV than in those who received raltitrexed. For the period up to week 10 of treatment, raltitrexed was consistently associated with significantly fewer toxicity-related symptoms than 5-FU + LV.

The data from the EuroQol questionnaire indicated that, at week 2 (i.e. during cycle 1), there was a highly significant difference in favour of raltitrexed in five of eight of the EuroQol dimensions (Figure 3). Patients receiving raltitrexed were approximately three times less likely to have problems with mobility and usual activities than those randomized to 5-FU + LV (odds ratio 2.90, $P = 0.0187$ for mobility; odds ratio 3.09, $P = 0.0022$ for usual activities). They were also at least twice as likely to have a better general health state (odds ratio 2.31, $P = 0.0025$), and there was a suggestion that they were also two to three times as capable of self-care as patients randomized to 5-FU + LV (odds ratio 2.49). However, this effect was not statistically significant ($P = 0.102$). In addition, at the same time point, the change from baseline in mean health state and index was statistically significantly better for patients receiving raltitrexed than for those receiving 5-FU + LV ($P = 0.0001$ and $P = 0.0455$ respectively). Subsequently, these differences appeared to diminish but there were still some statistically non-significant trends in favour of raltitrexed on the EuroQol scale and in total symptom advantages that were maintained to week 10.

Those differences in the week 2 quality-of-life assessments in favour of raltitrexed were perhaps not surprising when toxicity is taken into account. Of the 5-FU + LV patients, 28.0% required dosage reductions at cycle 2 because of toxicity. In contrast, only 4.4% of patients who received raltitrexed needed dosage reduction (Figure 4). There was a statistically lower incidence of WHO grade 3 and 4 mucositis in the raltitrexed than in the 5-FU + LV group, with less WHO grade 3 and 4 diarrhoea and leucopenia with raltitrexed. Liver transaminase rises were seen only in the raltitrexed group, but these rises were of limited significance as they were usually asymptomatic and self-limiting. The incidence of all other WHO grade 3 and 4 toxicities was similar for both treatments. The mean toxicity-related symptom scores at treatment weeks 2, 5 and 10 are shown in Figure 5.

Surrogate end points for quality of life have also been collected. Performance status of patients at study entry was documented. WHO performance status was 0 in 121 (49.0%) patients receiving raltitrexed and 106 (42.7%) of those receiving 5-FU + LV; 102 patients (41.3%) in the raltitrexed group and 125 patients (50.4%) in the 5-FU + LV group had a performance status of 1; the rest had a performance status of 2. Of patients with a baseline performance status of 1 or 2, 37.4% of those who received raltitrexed and 31.8% of those who received 5-FU + LV showed an improvement in performance status (odds ratio 1.28, $P = 0.42$). Weight gain occurred in 13% of raltitrexed and 18.9% of 5-FU patients. Disease-related symptoms improved in 86.1% and 83.1% of patients in the raltitrexed ($n = 115$) and 5-FU ($n = 124$) groups respectively.

© Cancer Research Campaign 1998

### DISCUSSION

Quality-of-life questionnaires must address disease-related symptoms and toxicities of the treatment under evaluation that have an impact on quality of life. The questionnaire must be completed frequently enough to detect difference, but should not be so much of an imposition that the patient declines altogether to complete it.

In study 3, the questionnaire was administered too infrequently to detect toxicity differences demonstrated early in the study. In addition, it did not reflect the main toxicities associated with treatment. Since the EORTC instrument was first issued, it has been updated and developed to include disease-specific modules (Aaronson et al, 1993). In the second study (study 12), the RSCL and EuroQol were administered early enough to detect at the time of the second course of therapy the significant differences in toxicity that had necessitated dosage reduction for 28.0% of patients treated with 5-FU + LV and 4.4% of those treated with raltitrexed.

Another problem concerns the timing of quality-of-life questionnaires in relation to the administration of treatment. The chemotherapy schedules in these studies were different, and the quality-of-life questionnaires were completed at different times in relation to chemotherapy administration and the onset of immediate and late side-effects. Quality-of-life instruments should be used early enough in the course of treatment to detect any differences in early toxicity. It is not clear with the tools available how to compare a treatment given on 1 day every 3 weeks with a regimen given for 5 consecutive days every 4–5 weeks.

The ideal frequency of quality-of-life questionnaire administration would be daily. Clearly, this would be a major imposition for the patient, but it may be possible to design a simple diary card that captures only data most liable to change. Gower and colleagues (1995) have used this approach to show short-term changes in quality of life that are related to acute symptoms induced by chemotherapy that would be missed by less frequently administered measures. They were able to identify a detrimental effect of a weekly regimen compared with a 3-weekly regimen, with patients preferring the 3-weekly regimen because of less nausea and vomiting.

The occurrence of missing data is a frequent problem in studies in which quality of life is being measured. The present studies could have been biased through mechanisms that result in loss of data (such as marked deterioration in quality of life in one or both of the treatment arms), although there do not appear to be serious biases in the results presented here. There are also the difficulties associated with the management of missing values within a given questionnaire: should the entire form be discounted if one value is missing, or can one or more values be imputed? What proportion of missing values is it acceptable to impute?

Tumour progression impacts on quality of life, with deterioration in quality of life becoming apparent, or useful quality-of-life data being lost because the patient is too ill to complete the questionnaire. The ways in which progression and death are handled with reference to quality-of-life analysis may also affect conclusions. In our analysis these factors were ignored.

Studies of quality of life may include a mixture of symptomatic and asymptomatic patients. Asymptomatic patients are more likely than those with symptoms to show reduced quality of life during a study because of their developing disease-related symptoms and/or signs of toxicity of therapy. Asymptomatic patients may unbalance a quality-of-life analysis if two groups, one of which contains more asymptomatic patients than the other, are compared. When designing protocols involving quality-of-life end points, consideration should be given to stratification of symptomatic and asymptomatic patients at study entry.

For many years clinicians have used performance status, changes in disease-related symptoms and toxicity to denote changes in patients' quality of life. In reviewing quality-of-life data from three major oncology journals, Batel-Copel et al (1997) concluded that, although performance status was an important measure, it was not adequate by itself to assess quality of life. There remains a requirement for a simple instrument that is convenient and easy for the patient to complete, but that measures performance status, changes in disease-related symptoms and toxicity, and the impact of these on the patient.

A tool to provide useful quality-of-life information in patients with advanced colorectal cancer is currently being piloted. The Chemotherapy Patient Monitor (CPM) consists of a checklist of the most clinically relevant toxicities affecting patients with advanced colorectal cancer, and gives an indication of the impact of treatment on patients. Patients are able to record the side-effects that they have experienced since their previous visit, indicating how troublesome these are and whether they wish to discuss them with their physician. Diary cards are also available to enable patients to make daily records between visits of side-effects. The CPM could prove useful in ensuring consistency of communication between patients and health professionals. It may also lead to more patient-centred treatment with improved patient satisfaction.

### ACKNOWLEDGEMENTS

This study was sponsored by a grant from Zeneca Pharmaceuticals and all analyses were performed by the Zeneca Biometrics Group.

### REFERENCES

Aaronson NK, Ahmedzai S, Bergman B, Bullinger M, Cull A, Duez NJ, Filiberti A, Flechtner H, Fleishman SB and de Haes JC (1993) The European Organization for Research and Treatment of Cancer QLQ-C30: a quality-of-life instrument for use in international clinical trials in oncology. *J Natl Cancer Inst* **85**: 365–376

Allen-Mersh TG, Earlam S, Fordy C, Abrams K and Houghton J (1994) Quality of life and survival with continuous hepatic-artery floxuridine infusion for colorectal liver metastases. *Lancet* **344**: 1255–1260

Batel-Copel LM, Kornblith AB, Batel PC and Holland JC (1997) Do oncologists have an increasing interest in the quality of life of their patients? A literature review of the last 15 years. *Eur J Cancer* **33**: 29–32

Bremnes RM, Andersen K and Wist EA (1995) Cancer patients, doctors and nurses vary in their willingness to undertake cancer chemotherapy. *Eur J Cancer* **31A**: 1955–1959

Cunningham D, Zalcberg JR, Rath U, Oliver I, van Cutsem E, Svensson C, Seitz JF, Harper P, Kerr D, Perez-Manger G and the Tomudex Colorectal Cancer Study Group (1996) Final results of a randomised trial comparing 'Tomudex'® (raltitrexed) with 5-fluorouracil plus leucovorin in advanced colorectal cancer. *Ann Oncol* **7**: 961–965

De Haes JCJM, Pruyn JFA and van Knippenberg FCE (1983) Klachtenlijst voor kankerpatiënten, eerste ervaringen. *Nederlands Tijdschrift voor Geneeskunde* **38**: 403–422

De Haes JCJM, van Knippenberg FCE and Neijt JP (1990) Measuring psychological and physical distress in cancer patients: structure and application of the Rotterdam Symptom Checklist. *Br J Cancer* **62**: 1034–1038

Fayers PM, Hopwood P, Harvey A, Girling DJ, Machin D and Stephens R (1997) Quality of life assessment in clinical trials – guidelines and a checklist for protocol writers: the UK Medical Research Council experience. *Eur J Cancer* **33**: 20–28

Glimelius B, Hoffman K, Graf W, Påhlman L and Sjödén PO (1994) Quality of life during chemotherapy in patients with symptomatic advanced colorectal cancer. *Cancer* **73**: 556–562

© Cancer Research Campaign 1998

Gower NH, Rudd RM, Ruiz de Elvira MC, Spiro SG, James LE, Harper PG and Souhami RL (1995) Assessment 'quality of life' using a daily diary card in a randomised trial of chemotherapy in small-cell lung cancer. *Ann Oncol* **6**: 575–580

Harper P, on behalf of the 'Tomudex' Study Group (1997) Advanced colorectal cancer (ACC): results from the latest Tomudex® (raltitrexed) comparative study (abstract 802). *Proc Am Soc Clin Oncol* **16**: 228a

International Working Group in Colorectal Cancer (1997) An international, multidisciplinary approach to the management of advanced colorectal cancer. *Eur J Surg Oncol* **23** (suppl. A): 4–66

Kerr DJ (1997) Clinical efficacy of 'Tomudex' (raltitrexed) in advanced colorectal cancer. *Anti-Cancer Drugs* **8** (suppl. 2): S11–S15

Nayfield SG, Ganz PA, Moinpour CM, Cella DF and Hailey BJ (1992) Report from a National Cancer Institute (USA) workshop on quality of life assessment in cancer clinical trials. *Qual Life Res* **1**: 203–210

Nordic Gastrointestinal Tumor Adjuvant Therapy Group (1992) Expectancy or primary chemotherapy in patients with advanced asymptomatic colorectal cancer: a randomized trial. *J Clin Oncol* **10**: 904–911

Scheithauer W, Rosen H, Kornek G-V, Sebesta C, Depisch D (1993) Randomised comparison of combination chemotherapy plus supportive care with supportive care alone in patients with metastatic colorectal cancer. *Br Med J* **306**: 752–755

Slevin ML, Stubbs L, Plant HJ, Wilson P, Gregory WM, Armes PJ and Downer SM (1990) Attitudes to chemotherapy: comparing views of patients with cancer with those of doctors, nurses, and general public. *Br Med J* **300**: 1458–1460

Taylor KM, Feldstein ML, Skeel RT, Pandya KJ, Ng P and Carbone PP (1994) Fundamental dilemmas of the randomized clinical trial process: results of a survey of the 1,737 Eastern Cooperative Oncology Group Investigators. *J Clin Oncol* **12**: 1796–1805

Williams A (1990) EuroQol – a new facility for the measurement of health related quality of life. The EuroQol group. *Health Policy* **16**: 199–208

*© Cancer Research Campaign 1998*